# Some of the Causes of Indigestion and Malnutrition in Nursing Infants

**Published:** 1893-07

**Authors:** Irving M. Snow

**Affiliations:** Buffalo, N. Y.; Lecturer on Diseases of Children, University of Buffalo; 476 Franklin Street


### Original Communications.

### SOME OF THE CAUSES OF INDIGESTION AND MALNUTRITION IN NURSING INFANTS.

By IRVING M. SNOW, M. D., Buffalo, N. Y.

Lecturer on Diseases of Children, University of Buffalo.

It is the experience of all physicians that occasionally breast-milk, the natural food of babies, may cause serious disturbances of digestion. When a nursing child fails to thrive, suffers much from colic or constipation, loses in weight, vomits, or has dyspeptic stools, we may attribute these symptoms almost certainly to poor or scanty breast-milk.

The object of this paper is to show the very close association between the health of the mother and the composition of her milk. If the nutrition of the mother is depressed from any cause, the various food elements in her milk become so altered in proportion that they will infallibly cause indigestion in her child.

To make an analogy: If you order a fare of cabbage and pork for a woman of sedentary habits, she will be apt to complain most bitterly of dyspepsia, and, in many instances, breast-milk is as unsuitable for a baby as are cabbage and pork for a delicate lady.

Now, indigestion in nursing children is worthy of our serious consideration. The infant may present such serious disturbances of the digestive and nervous system, that a change in diet may seem to be an urgent necessity. All of you will agree that giving a dyspeptic baby an artificial food is an exceedingly undesirable thing. The most scientifically prepared mixture of cow's milk, or of any dextrine food, is far inferior to human milk.

It is my opinion, that in a considerable number of cases weaning may be avoided, or be postponed by improving the mother's health, and thereby transforming the unnatural secretion of her breasts into normal milk. Here it is necessary to speak briefly of the physiology of lactation: Human milk contains virtually the same food elements as the dietary of an adult. There is a large proportion of water—eighty-eight per cent.—about four per cent. of fats, easily assimilated, potential in the formation of heat, the undigested residue acting as a natural laxative. We find from one to two per cent. of complex albuminoids, six to seven per cent. of a carbo-hydrate sugar of milk, and a minute quantity of mineral salts.

Of the food elements of human milk, the albuminoids are the most variable, the fats are next, and the lactose least of all. The more nearly the mother approaches an ideal standard of health, the more closely will the albuminoids approach two per cent. and the fats four per cent. of the total bulk. Both are elaborated by the metabolism of protoplasm in the cells of the mammary gland, all of the food elements, fats, albuminoids, and sugar are chiefly formed from proteid food. A civilized woman should live principally upon a fare of meat, poultry, and vegetable proteids, to secure a normal milk. If she be given large quantities of fatty food to remedy any deficiency of cream in her milk, she will probably develop an indigestion, and the fats in her milk will notably diminish.

In amount, the quantity of milk varies from one to three pints daily, increasing with the age and necessities of the child. A great advantage of lactation is that a baby suckled by its mother is seldom overfed. The position which the infant takes in nursing, and the slight effort and subsequent fatigue required to empty the collapsing gland, tend to prevent overfilling the stomach.

Yet this marvelous adjustment of human milk to the baby's needs is often disturbed. The composition of the milk is an index of the health of the mother. If she be ill, exhausted, or anemic, a marked change takes place in the milk. The percentage of albuminoids becomes larger, and the fats are diminished.

Now, in the infantile economy, the fats appear to be most essential and most easily assimilated, the proteids are less necessary, and when present in any excess, are digested with difficulty, causing pain, vomiting, and diarrhea.

Frequent nursing may also cause a change in the solid constituents of milk. The fats and albuminoids may be thus increased from twenty-five to fifty per cent. above their normal limits. Among the higher classes, the anemia, which haunts the Anglo-American woman, undoubtedly has an important effect in producing poor milk. As milk is elaborated from the blood, how can a normal secretion be made from poorly oxygenated blood? Lastly, in civilized woman, the duration of lactation is often considerably shortened ; a certain number of mothers have little or no milk after the fifth or sixth month. All of these facts explain much of the dyspepsias and malnutrition of sucklings.

As practical physicians, we judge of the good or bad qualities of breast-milk by the baby's appearance and behavior. The child should be plump and good-humored, should gain continually in weight, and should have from one to three semi-fluid stools a day, containing no undigested casein. To form a correct idea of an infant's health, it should be weighed once a week, the stools should be examined for undigested milk, and the cause of attacks of colic and restless nights should be carefully investigated.

In speaking directly of indigestion and malnutrition in nursing babies, it is convenient to first discuss—

1. Those cases in which the supply of breast-milk is merely inadequate for the infant's needs.

2. Those in which there is abundant milk, but where the child is irritable, dyspeptic, and losing weight, showing that the milk is unsuitable for its digestion.

Breast-milk is often secreted in very small amounts in the first weeks of lactation. In a certain number of cases, relatively small, I believe this condition may be due to imperfect development of the mammary glandular apparatus, but in a large number of cases, a scanty supply of milk is caused by improper hygiene, unwholesome diet, lack of out-of-door exercise, overwork, late hours, worry, insomnia, anemia, constipation, or too lengthened intervals of nursing.

The early failure of the milk may be due to an inadequate diet, directly after confinement, or there may be septic processes in the pelvis, the result of injuries to the parturient canal. The baby should be given the breast at proper intervals for at least two weeks, for the secretion of milk may be delayed. Too little persistence is usually shown. The attendants are entirely too ready to resort to artificial feeding.

Sore nipples are not always a valid reason for weaning. It may, however, happen in the first few weeks or months of lactation, that, although the baby is regularly nursed, it is not well, and the mother may be led to believe that her milk is too scanty or that it is unfit for the child's digestion.

The signs of an inadequate milk supply are :

1. The child will nurse for a long time, perhaps a half hour, before it is satisfied ; generally the breasts are emptied in ten to fifteen minutes.

2. The child will cry as if hungry, after nursing, is apt to be peevish and restless.

3. A poorly nourished baby is often plagued with wind-colic, the result of insufficient food. It is commonly constipated, having a small hard stool every two or three days.

4. The baby is weak, cold, languid, has flabby muscles. It has a stationary or diminishing weight.

Now, this phase of infantile atrophy is of frequent occurrence in the easy classes of society, and is a very serious thing if it take place in summer. When a physician finds the milk supply failing, and the baby in consequence deteriorating in health, he should carefully question the mother as to how she eats and sleeps, whether constipation is present, as to the amount of household work she does, and whether she takes a daily walk. Very many times the breast-milk may be increased up to the needs of the child by requiring the mother to go out daily into the open air, and by the avoidance of exhausting domestic duties.

To illustrate how a woman in fair health may from faulty hygiene have a very scanty supply of milk, and how, by the adoption of a proper regimen of diet and exercise, the amount of breast milk may be increased up to the needs of the child, I will relate the history of a case :

The mother was a strong, healthy, young, primapara. The child, a boy, was born after a normal labor ; weight at birth, seven and one-fourth pounds, including one pound of clothes. At first the baby was, with difficulty, induced to take the breast; after awhile it was nursed at intervals of two hours. I saw the child when it was four weeks old. Its weight was still seven and one-fourth pounds, but the mother declared that it was thinner than when it was born. The baby certainly presented an exceedingly atrophic appearance. The arms, legs, and body were wasted and wrinkled; the skin was cold and livid: temperature, normal. There was a foul-smelling stool after using a gluten suppository every two or three days. The baby was exceedingly fretful; it vomited occasionally, suffered a good deal from colic, and had worn the mother out by crying night and day.

My patient told me that she nursed the baby every two hours. The baby would usually hold the breast for half an hour, and then was seldom satisfied. She said that her breasts contained but little milk, and that, in her opinion, the child was half-starved. Her nipples were not sore; she was constipated and suffered from dyspepsia; she had not been out-of-doors for five weeks, although previous to her confinement she had led an active out-of-door life. It was evident that the baby's condition was due to lack of food, and that the mother's unhygienic habits had diminished her breast-milk. I directed her to take a daily walk; if possible, to go out twice a day; to nurse the baby every two and one-half hours for ten minutes, and afterwards to give the baby a weak cream mixture in a bottle, of which it might take as much as it wanted. My idea was to have the breasts regularly emptied, and to give the baby enough of the cream mixture to satisfy its appetite. As a result of this procedure, the child soon ceased to cry, the colic and constipation disappeared, and the baby rapidly grew plump and strong, and in nine weeks had gained five pounds. I visited the mother from time to time, and she reported that her health was greatly improved, and that her breast-milk was much increased in amount. January 2, 1892, she reported that the child would only take two or three ounces of the cream mixture a day, for her milk was almost sufficient for the baby's needs; she certainly had three times as much milk as nine weeks before.

The regular emptying of the breasts by the baby had supplied the physiological stimulus to the mammary glandular apparatus, and daily walks in the fresh, sunny autumnal air had so revived the mother's health, that in a relatively short time the secretion of milk nearly equaled the baby's wants. At the seventh month of lactation, the milk diminished, and the child was given a cream mixture.

The value of a correct hygiene and of regular out-of-door exercise in increasing a scanty milk supply has been demonstrated by Dr. T. M. Rotch, in an article on the Management of Human Breast Milk in Cases of Difficult Infantile Digestion (*Archives of Pediatrics*, November, 1890).

If it will be found impossible to bring the secretion of milk up to the needs of the baby, the child should be given in addition a small quantity of artificial food, but under no circumstances should it be entirely weaned. The baby may be allowed to nurse five or six minutes, and then should be given enough of a weak cream mixture to satisfy it. All of the breast-milk should be utilized ; even a small quantity of human milk will assist in the digestion of a subsequent feeding of cow's milk.

Another phase of infantile indigestion may occur when the secretion of milk is so copious that it oozes from the breasts in the intervals of nursing. In this class of cases, although there is enough milk, still the baby will not thrive. It may be very fretful, cry with frequent attacks of colic, will often vomit or pass lumps of undigested casein in the stools ; the child may even have convulsive or feverish attacks. The deterioration of the health in children may be so rapid that in two or three weeks they may present grave symptoms of atrophy.

When a suckling presents several or all of these symptoms, we may correctly infer that the milk is so charged, either from maternal ill-health or faulty hygiene, that it is unfit for the baby's digestion. If the breast-milk be analyzed, marked changes in the proportion of fats and albuminoids will be found; the albuminoids will not only be increased in amount and changed in character, but the percentage of cream will be lessened. An infant will generally show signs of dyspepsia and malnutrition when the albuminoids are more than two and one-half per cent., and the fats are less than three per cent. Casein, whether human or bovine, when present in excess, will cause colic by its slow, painful digestion ; often there is persistent vomiting.

Hence, when a nursing child is seriously disturbed in health, it is expedient to make a thorough examination of the mother and wet-nurse, and to direct a chemical analysis of her milk, and to question her closely as to her health and the routine of her life. Even so insignificant a matter as tight shoes may so harass a woman that her milk is unfit for her baby to digest. (See analysis, 8, 9, and 10.)

I have here a chart showing—

(1) The condition of the mother ; (2) Analysis of her milk ; (3) Health of the child.

Many of the cases are taken from Rotch's paper, before alluded to.

| | 1 | 2 | 3 | 4 | 5 | 6 | 7 | 8 | 9 | 10 | 1 | 2 | 3 | 4 |
|---|---|---|---|---|---|---|---|---|---|---|---|---|---|---|
| | Normal milk. | Mother, puerperal eclampsia; albuminuria, one-fourth per cent.; child ill. | Same case; breasts regularly pumped; out-of-door life; trace of albuminuria; child well. | Effect of mental anxiety; child ill. | Mother menstruating; child restless. | Milk of a wet-nurse taking too rich food; child vomiting curds. | Milk of a pregnant woman; child ill. | Mother eating heartily, no exercise; child suffering from colic and vomiting. | Mother taking exercise; feet sore from tight French shoes; child ill | Mother walking two miles a day in easy shoes; child well. | Mother weak, suffering from dyspepsia and headaches; child colic, vomiting curds, curds in stools. | Mother improved in health; baby no colic, only occasional regurgitation of milk. | Mother suffering from loss of sleep and anxiety from nursing a sick baby; mother with a bloody uterine discharge. | Milk of a woman with cracked nipples; baby colic, curds in stools, losing flesh. |
| Water ......... | 88. | 88.51 | 87.70 | 89.17 | 89.60 | 83.50 | 89.61 | 86.80 | 90.10 | 87.59 | 88.95 | 87.20 | 89.37 | 87.60 |
| Solids ........ | 12. | 10.49 | 12.30 | 10.83 | 10.40 | 16.50 | 10.39 | 13.20 | 9-90 | 12.41 | 11.05 | 12.80 | 10.63 | 12.40 |
| Fat ........ | 3-4.0 | 1.62 | 3.20 | .62 | 1.37 | 5.44 | 0.80 | 3.05 | .65 | 3.34 | 1.83 | 3.62 | 1.90 | 3.18 |
| Albuminoids .. | 1-2.0 | 3.54 | 2.52 | 4.21 | 2.78 | 4.61 | 4.50 | 3.89 | 3.82 | 2.61 | 4.77 | 2 44 | 1.75 | 3-53 |
| Sugar ......... | 6-7.0 | 6.10 | 6.40 | 5.80 | 6.10 | 6.25 | 5.00 | 6.10 | 5.25 | 6.30 | 5.24 | 6.49 | 6.81 | 5.72 |
| Ash ........... | 00-2 | 0.17 | 0.18 | 0.20 | 0.15 | 0.20 | 0.09 | 0.16 | 0.18 | 0.16 | 0.20 | 0 26 | 0.17 | 0.22 |
| | | Rotch. | Rotch. | Rotch. | Rotch. | Rotch. | Rotch. | Rotch. | Rotch. | Rotch. | Snow. | Snow. | Snow. | Snow. |

Below will be found a series of exceptional cases selected from a large number of analyses, illustrating peculiarities of infantile digestion. Infants all digesting well and gaining in weight.

HUMAN BREAST-MILK ANALYSIS.—ROTCH.

| | 1 | 2 | 3 | 4 | 5 | 6 | 7 | 8 |
|---|---|---|---|---|---|---|---|---|
| Fat | 4.37 | 3.76 | 3.16 | 3.82 | 2.96 | 2.09 | 2.02 | 2.36 |
| Milk Sugar | 6.30 | 6.95 | 7.20 | 5.70 | 5.78 | 6.70 | 6.55 | 7.10 |
| Albuminoids | 3.27 | 2.04 | 1.65 | 1.08 | 1.91 | 1.38 | 2.12 | 2.20 |
| Ash | 0.16 | 0.14 | 0.21 | 0.20 | 0.12 | 0.15 | 0.15 | 0.16 |
| Water | 85.90 | 87 11 | 87.78 | 89.20 | 89.23 | 89.68 | 89.16 | 88.18 |

These tables are most interesting, for they not only explain the cause of infantile indigestion, but demonstrate that an improvement may often be obtained after the adoption of a proper regimen. As a matter of fact, the treatment of these is principally hygienic, the troubles caused by menstruation or a cracked nipple soon pass away. The woman should lead a placid, comfortable life. She should be relieved from excessive household work. To transform poor milk into good, to decrease the undigestible abuminoids, and to increase the fats, no means is better than daily walks (below the point of fatigue) in the open air. The tonic effect of out-of-door exercise is more efficient than any medication at our command. A very important problem is the feeding of the baby while the milk varies from the normal standard. I will say that when you have secured a proper hygiene for the mother, a rapid improvement usually takes place. If the baby be not acutely ill, it may certainly take some of the breast-milk. It will usually digest the milk ; or may be allowed to nurse four or five minutes, then be given a few teaspoonfuls of sterilized water, then be allowed to satisfy itself at the breast. The breast-milk is thus diluted in the baby's stomach.

To illustrate the causes of indigestion in a suckling, and also to show the improvement following good maternal hygiene, the following case is of value :

The child, a girl of six weeks, was the offspring of a healthy Anglo-American mother. Child was nursed every two hours. The mother had so much milk that it oozed from her breasts; nevertheless, the baby had been very fretful for two weeks. It was plump and of good color ; it occasionally vomited after feeding ; one stool a day. The baby seemed in great distress most of the time ; it had cried all night and all of the daytime for two weeks. An examination of the child's ears, throat, skin, chest and abdomen, showed nothing out of the way. On closely questioning the mother, I found that she had not had an undisturbed night's sleep for a long time; also that she was constipated, had a stool every two to three days; that she had a cracked nipple, and when the baby nursed she suffered agonizing pain. Finally, she had not been out of the house for six weeks.

My idea of the case was that the baby was plagued with indigestion from an excess of albuminoids in the milk, and that this altered milk was due to a sore nipple and lack of fresh air. The mother was directed to nurse the baby for five minutes, and then to give it four to five teaspoonfuls of sterilized water, and then to allow child to have all the breast-milk it wanted. The sore nipple was treated and the mother took daily walks, and was much improved in health. Almost immediately the baby ceased to cry, and grew good-humored again. After a week the mother stopped diluting the milk. I strove in vain to obtain a sample of milk for analysis. After the baby was better the chemist received a sample, whose analysis showed a correct proportion of fats and albuminoids.

The next case shows how maternal dyspepsia and debility may cause the secretion of milk difficult of digestion:

Baby, weight at birth, seven and a half pounds, four weeks old, suckled by Anglo-American mother. Since its birth the child had been very fretful, crying night and day. It vomited curdled milk after each nursing, had six or seven stools a day; yellowish, stiff with undigested casein, frequent attacks of colic; nevertheless, the baby was plump and strong, showing good powers of digestion to assimilate its unwholesome food. There was slight rachitic beading of the ribs; the thighs and perineum were red and excoriated.

Condition of the mother: Age, thirty-eight; only moderate strength and vitality; has convalesced rapidly since confinement; has scarcely been out of doors for five weeks; is easily tired, and has for years been tormented with flatulent dyspepsia. Associating the dyspepsia of the child with that of the mother, I suggested an examination of the milk, and found (see first table, No. 1,) an excessive amount of casein. The cause of the indigestion was evident. The mother was given Hcl. and peptonoids, and advised to walk or ride daily. During the following three weeks both mother and child were better, although twice, for a couple of days, the baby had numerous diarrheal stools. On July 26th, the milk was again examined, showing a remarkable improvement. (See first table, No. 2.) The mother was nearly relieved of her dyspepsia and felt rested. The child now rarely had attacks of colic; the stools showed no undigested milk; the condition of the child was fairly satisfactory, although the percentage of casein was too great for easy digestion. I regarded it as a great achievement to carry the baby through the hot season without weaning or serious illness. In October I again saw the baby; it had gained in weight; did not vomit; it, however, had frequent attacks of colic, and occasionally passed undigested casein in its stools.

The mother was unable to take much exercise, having very exacting domestic duties. Her dyspepsia had returned, and she was harassed by headaches. As the hot season was past and the baby's digestive organs were relatively mature, I advised weaning and the administration of a weak cream mixture. My little patient again became good-humored, having no longer an excessive quantity of albuminoid to digest.

This case illustrates why a child receiving an abundant supply of breast-milk may be afflicted with indigestion; also how quickly the fats and albuminoids in human milk may approach a normal standard with improved maternal health; and, lastly, how quickly the milk may again deteriorate with recurring ill-health.

A third case will show how pain from a cracked, deformed nipple will alter milk:

Mother, Anglo-American, had been unable to nurse her first child. With the second she had a long difficult instrumental labor. I saw the baby when it was four weeks old; child emaciated, fretful, passing curds in its stools, analysis of milk (See first table, No. 4.) Mother had a painful, cracked nipple. The milk was insufficient in quantity and absolutely unfit for the child's nutrition. All efforts to improve the milk failed, I think, on account of deformity in the nipple. The act of nursing produced such intense pain, that the breast-milk was altered in character. Child weaned and reared on a weak cream mixture.

I am well aware that this paper does not discuss all of the causes of indigestion in nurselings, nor does it relate a series of brilliant cures. Any assertion that poor milk can be changed into good milk would be strongly charged with falsehood. In conclusion, I would say that dyspepsia in sucklings receives too little thought and attention from the profession. Illness in a nursing woman means poor milk and a sickly baby. The foundations of constitutional weakness and life-long dyspepsia may in this way be laid. Very often skilful treatment of the mother will relieve both mother and child. Surely, no epoch in a woman's life is more important than that when her own well-being is associated with that of her child.

476 Franklin Street.

---

The Belgian Academy of Medicine has offered a prize of 4,000 francs ($800) for the best essay upon the pathology and treatment of epilepsy.

